# Dual pathways of concealed gun carrying and use from adolescence to adulthood over a 25-year era of change

**DOI:** 10.1126/sciadv.adp8915

**Published:** 2024-12-04

**Authors:** Charles C. Lanfear, David S. Kirk, Robert J. Sampson

**Affiliations:** ^1^ Institute of Criminology, University of Cambridge, Cambridge CB3 9DA, UK.; ^2^ Department of Criminology, University of Pennsylvania, Philadelphia, PA 19104, USA.; ^3^ Department of Sociology, Harvard University, Cambridge, MA 02138, USA.; ^4^ American Bar Foundation, 750 North Lake Shore Drive, Fl. 4, Chicago, IL 60611-4557, USA.

## Abstract

Most homicides in the United States are committed using a handgun, but little research examines gun carrying over critical stages of the life course and changing contexts of violence. Notably, although most of the handgun homicides are committed by adults, most research on concealed gun carrying focuses on adolescents in single cohort studies. Using more than 25 years of longitudinal multicohort data from Chicago, 1994–2021, we show that pathways of concealed gun carrying are distinct between adolescence and adulthood. Adolescent carrying is often age-limited and responsive to direct exposure to gun violence (witnessing and victimization), while adult carrying is a persistent behavior that is less tied to direct exposure. The onset of concealed carry is also a strong predictor of later gun use (shooting or brandishing), and we find distinct patterns of gun use between individuals who first carry in adolescence versus adulthood. We discuss the implications of these dual pathways for research and policies on firearm use.

## Teaser

Distinct patterns by life-course stage—adolescence versus adulthood—reveal dual pathways for concealed gun carrying and gun use.

## INTRODUCTION

**Fig. 1.**
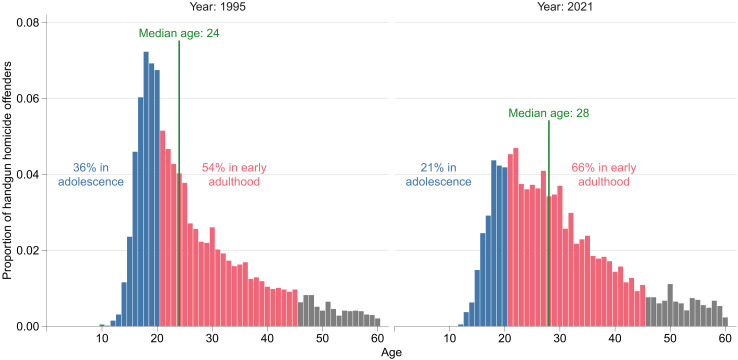
Proportion of handgun homicide offenders by recorded age in 1995 and 2021 according to FBI Uniform Crime Report Supplementary Homicide Reports. The 3.5% of homicide offenders that are over age 60 are omitted. For the 15% of firearm homicides with more than one recorded offender, the age of the first offender was taken. If limited to the 85% of single offender homicides, then an even larger proportion of homicides is attributable to adults.

A known fact is that most homicides in the United States are committed with a firearm, and of those, the vast majority are committed with a handgun. Between 1995 and 2021, for example, 89% of homicides with a known firearm were committed using a handgun (*1*). Less appreciated is that, although most research on concealed gun carrying focuses on adolescents (*2*), most handgun homicide offenders are in fact adults (*1*). A focus on adolescents may have been warranted by age patterns of gun violence in the 1990s or earlier, but recent increases in gun violence have been concentrated among adults. Figure 1 confirms a marked shift in age-specific patterns of homicide over time: Offenders are now far less likely to be in adolescence, with the median age of homicide offenders increasing a full 4 years from 1995 to 2021. This time period coincides with the widespread shift toward “shall issue” concealed carry laws and, more recently, permitless carry laws, with research revealing that this deregulation of concealed carry laws was associated with subsequent increases in homicide (*3*).

Not only do we know little about the course of gun carrying during and after adolescence, but we also know little about age-graded pathways of gun carrying across important periods of contemporary social change. For example, the stock of firearms in the United States has doubled over the past quarter century (*4*), and the spike in homicide rates throughout the country in 2020 has been attributed to a surge in firearm sales—and presumably gun carrying—following the outbreak of COVID-19 and protests against racialized police violence (*5*–*7*). Data from the Bureau of Alcohol, Tobacco, Firearms and Explosives (ATF) further reveal that the time between purchase of new guns and the use of these guns in a crime declined in the wake of the 2020 surge in gun sales, suggesting that COVID-era gun purchases were increasingly used in the commission of crimes (*8*). Yet, as is well known, age and historical change are confounded in single cohort studies, making it challenging to distinguish their independent effects (*9*). Longitudinal, multicohort data are necessary to reveal and explain changing age-related patterns of gun carrying and use in the United States.

This paper directly addresses these gaps in our scientific knowledge on the life course of concealed gun carrying and use from adolescence through adulthood by analyzing 25 years of longitudinal multicohort data from the Project on Human Development in Chicago Neighborhoods (PHDCN+). Concealed gun carrying refers to the practice of carrying a gun in public that is concealed, or hidden, from the view of others, whether legally or illegally. With our research design, we are able to analyze patterns of concealed gun carrying by sex, race, cohort, and exposure to violence from childhood through adulthood, specifically: (i) at what age onset of concealed carrying occurs; (ii) if the age of carry onset is predictive of recent gun carrying; (iii) if an earlier age of carry onset is predictive of *using* a gun in a crime or for self-defense; and (iv) if exposure to gun violence predicts onset of carry for both juveniles and early adults. And whereas much of the literature on concealed carry focuses on either illegal or legal carrying, a contribution of our study is to focus on both (*10*–*12*), including the differing pathways to each type of behavior.

As our primary focus is on exploring differences in concealed carry onset by age, the design of the PHDCN+ is uniquely well suited to examine these patterns as it includes a quarter century of follow-ups of a diverse and representative sample of four age cohorts of Chicago children, born from approximately 1981 to 1996, who experienced different contexts of gun violence and criminal justice contact at different stages of life (*13*). For example, the oldest cohorts came of age during the violent crime peak of the early 1990s and the youngest reached their early 20s during spikes in gun violence starting in 2016, whereas the middle cohort reached their teens during an era of relatively low crime and violence (*14*).

The ensuing analysis will show that concealed gun carrying is responsive to these surges in violence in Chicago, which mirrored patterns of gun violence seen in major cities across the United States (*15*). Yet, the analysis will also show that patterns of concealed gun carrying are to an important extent distinct between adolescence and adulthood: Adolescent carrying is less persistent but is responsive to direct exposure to gun violence through victimization or witnessing shootings, whereas adult carrying is a more persistent behavior that is less tied to intimate exposure to gun violence. We refer to these distinct patterns by life course stage—adolescence versus adulthood—as a dual pathway explanation for concealed gun carrying.

This longitudinal study is based on a representative sample of children originally living in Chicago, 82% of whom were living in the Chicago metro area in the most recent survey wave in 2021. This may affect the generalizability of our results if Chicago or Illinois are an unusual context for concealed gun carrying or gun violence. However, rates of gun homicide in Chicago over the study period were similar to those in other major US cities such as Philadelphia and Dallas and substantially below the most violent cities in America such as St. Louis, Baton Rouge, New Orleans, Memphis, and Baltimore (*1*). Gun laws in Illinois, reputed to be some of the toughest in the country in the late 20^th^ and early 21^st^ centuries, also became permissively similar to other states in 2014, as many of our sample members reached the legal age of carrying a firearm. Our multi-cohort design thus allows us to compare patterns of concealed gun carrying at the same ages, in a context where carrying would have been illegal in Illinois for older cohorts but legal for younger cohorts.

### Concealed carry

Until 2013, concealed carrying of firearms was banned in Illinois, yet the ban was ultimately ruled unconstitutional under the Second Amendment. To address this ruling, the Illinois’ Firearm Concealed Carry Act was enacted on 9 July 2013, with permits issued starting in 2014, allowing for concealed carry in Illinois with a license. With the enactment, Illinois became a “shall-issue” state, meaning that the state must issue concealed carry licenses to applicants who satisfy eligibility requirements. At the time, nearly every US state required individuals to have a permit to carry a concealed gun, under either a shall-issue policy such as in Illinois or a “may-issue” policy in which the applicant needed to demonstrate a need for carrying a concealed firearm (*16*).

The landscape of concealed carry laws nationwide changed markedly in the ensuing decade, particularly in the case of *New York State Rifle & Pistol Association Inc.* v. *Bruen* (2022), in which the US Supreme Court ruled that New York’s may-issue concealed carry law, the 1911 Sullivan Act, was unconstitutional. The law had required concealed carry permit applicants to show “proper cause” as to why they would need to carry a weapon in public, with the government holding discretion to reject applications for a license. The Supreme Court ruled that the law prevented individuals from exercising their Second Amendment rights, and this ruling had the effect of making concealed carry in New York and elsewhere a presumptive right.

While concealed carry is a right throughout the United States—in some jurisdictions requiring a permit—credible estimates on the extent to which individuals have exercised that right remain elusive. The only official source of carry statistics comes from counts of issued permits. These only indirectly measure rates of carry, as many individuals carry illegally, those with permits may not carry, and 29 states, as of this writing, do not require permits to concealed carry. Accordingly, survey information on gun carrying is advantageous for overcoming the limitations of gun permit data. For instance, a 2017 Pew survey estimated 22% of Americans own a handgun, and of those, 57% carry a handgun outside the home at least some of the time (*17*). Similarly, estimates from the 2019 National Firearms Survey indicate 30.3% of American handgun owners carried in the past month, and 38.1% of those did so every day (*18*). Concealed gun carrying has likely increased since these estimates due to unprecedented increases in gun purchases in 2020 following the outbreak of COVID-19 and protests against racialized police violence (*6*).

What little we know about the prevalence of concealed carry, however, tells us almost nothing about patterns over the life course, such as when people start and discontinue carrying. In one of the few analyses of concealed carry over the life course, Comer and Connolly (*19*) reported a relatively stable rate of 5% of National Longitudinal Survey of Youth 1997 respondents—born from 1980 to 1984, paralleling the ages of our three oldest cohorts—having carried a handgun in the past year between 1998 and 2011 (i.e., to approximately age 30). This rate of carry was stable despite rates of gun violence dropping precipitously nationwide and respondents only becoming eligible to legally carry concealed weapons (i.e., reaching age 21) mid-way through the analysis period. The interyear correlation in carrying increased steadily with time, however, indicating stronger continuity in carry among adults than adolescents [page 5 in (*19*)].

The motivations for carrying a concealed gun likely differ across the life course. Protection of selves and family is the most commonly stated reason for carrying a concealed firearm, regardless of age (*2*, *10*, *11*, *20*–*22*). However, there is reason to hypothesize that the reasons why, or from whom, individuals desire protection will differ across stages of life. First and foremost, gun carry is illegal for adolescents, so gun carry is by definition delinquent behavior. Gun carry in adolescence is often associated with living in dangerous circumstances (*21*)―e.g., being exposed to dangerous peers due to gang membership and involvement in drug markets (*23*, *24*) or living in neighborhoods perceived as unsafe and lacking in social control capacity (*12*). These circumstances increase the likelihood of being personally exposed to gun violence, which has been implicated as a key proximate cause of adolescent gun carrying and subsequent gun violence (*25*–*27*). Exposure to gun violence is pervasive in the United States, particularly during adolescence, but prevalence differs starkly by race, sex, and birth cohort, and these differences—particularly by race—grow throughout adulthood (*14*).

The relationship between exposure and gun carrying appears strongest for youth with histories of delinquency (*26*, *27*). Using data from the Pathways to Desistance study of adjudicated adolescent offenders, Beardslee *et al.* (*28*) found that exposure to gun violence—but not other forms of violence—increased the probability of gun carrying during the mid-teens through mid-twenties. While the oldest three cohorts of the PHDCN+ data we use here were born in the same period as the Pathways to Desistance respondents, the PHDCN+ sample is representative of the population of Chicago’s children in the 1990s, rather than just those adjudicated for a criminal arrest. Using earlier waves of the PHDCN+, Bingenheimer *et al.* (*25*) found exposure to firearm violence approximately doubled the likelihood of carrying a concealed weapon or engaging in serious violence. Carry was not separated as an outcome from commission of violence, however, and respondents were only followed for about 5 to 6 years. As a result, the oldest respondents were only observed through age 21 at most.

Less is known about how proximate exposure to gun violence relates to adult gun carrying, whether legal or illegal. While protection is often cited as a primary motivation for adult gun ownership, paradoxically rates of gun ownership are high among groups less likely to be the victims of violence, e.g., those older, white, and rural, and low among groups more likely to be exposed to violence, e.g., those young, Black, and urban (*14*, *17*); the same can be said for carrying rates among white and Black Americans (*29*). However, among adult gun owners, carriers tend to be younger and more often male (*18*). Comer and Connolly (*19*) found that exposure to gun violence before the age of 12 was associated with higher rates of past-year gun carrying in adolescence, but this association weakened through early adulthood (observed to age 30). Their insightful analysis, however, does not examine the effect of direct exposure to gun violence during the early years of adolescence (i.e., age 12 to 15) when exposure rates tend to accelerate markedly (*14*).

While proximate exposure to violence has been found to be a primary predictor of gun carrying among adolescents, there is some evidence that increases in gun ownership and concealed gun carrying among adults may instead be tied to political orientation and perceptions of generalized danger rather than immediate dangerous circumstances. This generalized danger includes the perceived inability of police to guarantee safety (*10*), an aspect of legal cynicism (*30*). This is one explanation given for the sharp rises in both sales and concealed carry permitting following the political polarization of recent presidential elections (*10*, *11*, *31*) and, more recently, the COVID-19 pandemic and the police murder of George Floyd (*5*, *6*, *32*). Many who purchased guns in response to the pandemic cited a general “lawlessness” as a reason (*33*).

We advance research on the relationship between exposure to violence and concealed gun carrying by testing whether the expected association between exposure to gun violence and onset of gun carrying in adolescence (up to age 21) is persistent and also observed in adulthood (up to age 34). In doing so, we also consider exposure to violence occurring as late as age 21. Before turning to this relationship, we examine the age of onset of concealed gun carrying broken down by race, sex, and age cohort. After establishing the key patterns in our data, we turn to an examination of whether an early onset of gun carrying is predictive of a stable pattern of carrying throughout one’s life course. Similarly, we examine the relationship between the age at which individuals first carry a gun and their likelihood of using a gun (i.e., for self-defense or otherwise). Together, these tests provide insights on the temporal processes and sources of gun carrying over the life course.

### Data

We analyze data from the Project in Human Development in Chicago Neighborhoods Longitudinal Cohort Study (PHDCN-LCS) that included a representative sample of 3403 Hispanic, non-Hispanic Black, and non-Hispanic white children in four age cohorts (infancy, aged 9, 12, and 15) drawn in the mid-1990s from a random sample of 80 of Chicago’s 343 neighborhoods stratified by racial-ethnic composition and socioeconomic status (*34*). These respondents were interviewed three times between 1995 and 2002. A random subsample from these four cohorts were selected for long-term follow-up and surveyed in 2012 (*N* = 1013, 63% response rate among the eligible subsample) and 2021 (*N* = 651, 66% response rate), including respondents who moved outside Chicago during the study period. At wave 5, 51% of respondents were living in Chicago and 82% in the wider Chicago metropolitan area. See figure S1 for a diagram of the study design. The Harvard University Institutional Review Board approved this study, and informed consent was obtained for survey data collection. Hereafter, we refer to the age cohorts by modal birth year to aid in recalling their historical contexts; for example, the cohort sampled at age 15 during the first wave of data collection in the mid-1990s is referred to as the 1981 cohort, as 65% of the cohort were born during that year (and the remainder in adjacent years).

For our analyses, we include all members of these cohorts who answered at least one question regarding onset of concealed gun carrying or gun use in any wave rather than limit analyses to just respondents from the fifth wave (see fig. S2). This results in analytical samples of 2121 for longitudinal analyses of gun carrying, 926 for longitudinal analyses of gun use, and 642 for past year analyses of gun carrying with the wave 5 sample. The difference in longitudinal and past year sample sizes primarily reflects study design rather than attrition, and the sample is still representative of individuals who were children in Chicago in the mid-1990s. Attrition rates are similar to other urban long-term follow-up designs (*35*), and below, we address attrition through weighting, although unweighted results are substantively equivalent. See fig. S2 for a diagram of the analytical samples, table S1 for a comparison of the generally modest baseline differences between the sample at study inception in 1994 (wave 1) and in 2021 (wave 5), and (*34*) for a detailed description of the research design of the PHDCN+.

Race and ethnicity as well as sex were reported by the respondent’s primary caregiver at wave 1. Race and ethnicity were combined into a single three category measure (“Hispanic,” non-Hispanic “Black,” and non-Hispanic “white”); while individuals of other races and ethnicities were included in data collection, they are excluded because the subsample was too small to conduct analyses of gun carrying and gun use (*N* = 85 across all waves, *N* = 31 in wave 5).

Age of onset of gun carrying was measured using questions from waves 2, 3, and 5 asking if respondents ever carried a concealed gun, when this last occurred, and the age it first occurred (see the Supplementary Materials for exact questions). Of the 2121 respondents in the longitudinal analysis sample, 199 (9.4%) reported ever carrying a concealed gun. To measure continuity in carrying into the present, recent concealed carry was measured with a question in wave 5 (i.e., 2021) asking respondents if they had carried a concealed gun in the past year. Past year analyses are restricted to the 642 wave 5 respondents, of whom 73 (11.4%) reported carrying in the past year. Those reporting past-year carry were also asked if they had a permit to do so. All wave 5 respondents who reported carrying a concealed gun in the past year resided in jurisdictions where a permit was required to carry a concealed gun in public. As a result, this variable is taken as an indicator of the legality of past-year carry.

Age of onset of gun use was measured using questions from waves 3 and 5 asking respondents how old they were when they first “used a gun, even if it was not fired, to protect yourself, someone else, or your property” or when they first “shot or shot at” someone (see the Supplementary Materials for exact questions). The sample size for the analysis of onset of gun use (*N* = 926) is smaller than that of concealed carry because only the 1981 cohort was asked about gun use in wave 3; all other cohorts were asked only in wave 5 (see fig. S1). Seventy-five (8.1%) of these respondents reported ever using a gun. One-quarter of those reporting any gun use reported both using a gun for defense and shooting/shooting at someone. For these respondents, the earlier age was taken as the age of onset of gun use. Whereas the raw count of gun use (*N* = 75) and concealed gun carrying (*N* = 199) among the respondents is relatively modest in size, we reiterate the unique advantage of our data to studying gun-related behavior from childhood through mid-adulthood, and in the ensuing analyses, we take care to show the level of uncertainty in our estimates. Even so, our analyses reveal previously unexamined findings about gun-related behavior over the life course, such as greater persistence in carry over the life course among individuals who started carrying in adulthood rather than in adolescence.

While in policy debates there is substantial interest in the effectiveness of concealed carry at reducing victimization through justified defensive gun use, when estimating prevalence of gun use, we do not distinguish between justified and unjustified gun use. This is because, even with the exacting level of detail in the PHDCN+ data, it is generally not possible with survey data to affirmatively identify instances of gun use that meet any legal criteria for self-defense. For example, nearly 30% of respondents reported that their first defensive gun uses occurred in situations where their target was neither committing a crime nor engaged in an argument or fight with the respondent. Even if the target was committing a crime, defensive gun use may still occur in unjustified circumstances—such as if the respondent initiated a fight—or while the respondent is also committing a crime—such as using a gun to prevent being robbed while dealing drugs. These and other related issues are inescapable limitations of survey self-reports for estimating the prevalence of legally justified defensive gun use (*36*).

Last, the age of first exposure to gun violence was measured using questions from waves 1, 2, 3, and 5 asking respondents if they had ever personally seen someone shot or were shot themselves, and if so, how old they were when it occurred. If both events occurred, then the earlier age was taken. Six hundred forty (30.2%) respondents reported exposure to gun violence. A set of individual and neighborhood characteristics are included as covariates in the exposure analyses: socioeconomic status, immigrant family, family history of arrest, and neighborhood homicide rate, percent population Black, percent of households living under the poverty line, and population density. Their measurement is described in the Supplementary Materials.

## RESULTS

**Fig. 2.**
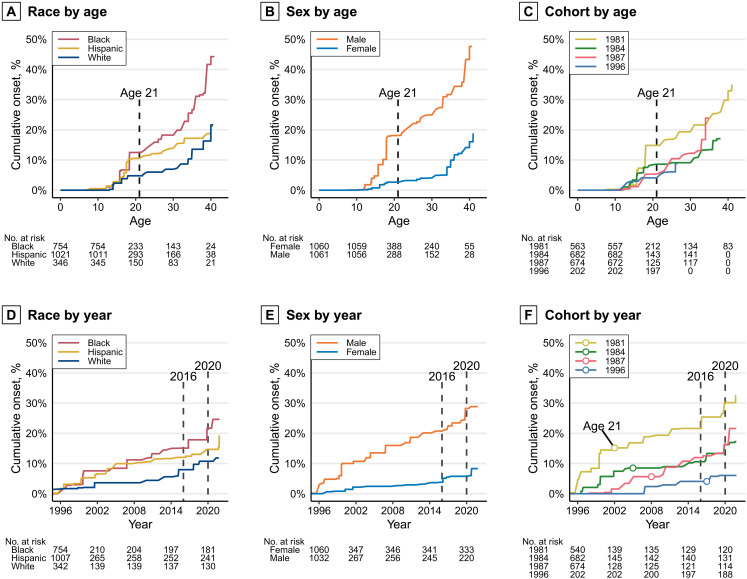
Nonparametric estimates of the cumulative probability of onset of concealed carry stratified by race, sex, and birth cohort. (**A** to **C**) Top row: Curves by respondent age. (**D** to **F**) Bottom row: Curves by year. In the cohort by year (F), a circle marks the mean 21st birthday of each cohort. We estimated these curves of concealed carry by age separately for race, sex, and birth cohort using Turnbull’s (*52*, *53*)nonparametric maximum-likelihood estimator (NPMLE).

By age 21, an estimated 10.2% [95% confidence interval (CI): 2.3 to 17.4] of our study population had carried a concealed gun, 14.4% by age 30 (95% CI: 6.3 to 21.8), and 31.9% by age 40 (95% CI: 17.9 to 43.6). Figure 2 unpacks this result and presents estimated cumulative probabilities of concealed carry by age (top row, Fig. 2, A to C) and calendar year (bottom row, Fig. 2, D to F) stratified by race, sex, and cohort. Displaying curves by both age and year allows for distinguishing between age-specific and period-specific changes in onset rates of concealed carry.

Looking first to age curves, regardless of stratification, all curves display a relatively similar age-specific shape: Onset of carry increases rapidly in the middle of adolescence (13 to 18), plateaus until 21, then gradually increases from 21 onward (Fig. 2, A to C). A more rapid increase is again seen near the end of each curve, but this is a period- rather than age-specific change, as we will show. Of all of the individuals who ever carried a concealed gun in our sample, roughly one-third first carried before the age of 21, which we refer to as adolescent-onset carrying, and two-thirds first carried from age 21 or later (adult onset). Given that the sample originated in Illinois and the nature of the gun laws in Illinois throughout the study period, we assume that all of the gun carrying in our sample before the age of 21 was illegal, and we will later show to what extent recent post-21 carrying was legal or otherwise.

Within the age curves, racial differences are substantial, with concealed carry by age 18 over twice as likely for Black and Hispanic than white individuals (Fig. 2A). From 21 to 40, the difference in the probabilities of having carried a concealed gun for white and Hispanic individuals remains before converging at 40. In contrast, the cumulative probability for Black respondents accelerates in the 30s to reach more than 40% by age 40. Sex differences are similarly pronounced, with male carry increasing rapidly in the late teens and then increasing relatively steadily through age 40 (Fig. 2B). Female carry is uncommon until age 35 when there is a rapid increase approaching 20% by age 40.

In the top right panel, we see the oldest cohort, those born in 1981, reporting the sharpest increase in carrying during the teen years, followed by those born in 1984 (Fig. 2C). The younger 1987 and 1996 cohorts have low and similar rates of onset before adulthood. In later life, however, cohort differences change markedly. In particular, onset for the 1987 cohort accelerates faster than the other cohorts in the mid-30s, coinciding with the COVID era, to reach parity with the 1981 cohort, which displayed the highest rates of onset in adolescence. Similarly, onset for the 1996 cohort reaches parity with the 1984 cohort at age 26.

Looking next to the year curves, we see that the increases at the tail of the age curves are due to an increase in onset of concealed carry within every cohort in 2016 through early 2021: After the initial rises from staggered periods of adolescence, the curves increase linearly until accelerating in approximately 2016 and, particularly, 2020 (Fig. 2, D to F). By 2021, an estimated 18.3% (95% CI: 10.5 to 25.3) of our study population had carried a concealed gun. These recent increases appear to represent cross-cutting period-specific effect on all older adults, a finding that prior research has not uncovered; no increase is observed among the youngest cohort (1996). This finding is consistent with national survey estimates that reveal that COVID-era gun buying, including by first-time buyers, was particularly pronounced among individuals in their 30s (*6*, *37*).

Differences between strata seen in the age curves are otherwise replicated in these year curves. For instance, whereas we observed that the cumulative probability of concealed carry by age 40 was more than 2.5 times greater for men compared to women, by the end of our study period in 2021, an estimated 28.9% (95% CI: 16.2 to 39.6) of males had carried a concealed gun compared to only 8.3% (95% CI: 2.5 to 13.8) of females (Fig. 2E). Note that the maximum probability for each year curve is necessarily lower than the maximum of the corresponding age curve because the year estimates were calculated from respondents of different ages, even within cohorts, and younger respondents experience less exposure time. That is why, for instance, the maximum cumulative probability seen for men in the age curves in the top of Fig. 2 (i.e., 47.6% by age 40) is different than the maximum cumulative probability for men by year 2021 (i.e., 28.9%).

The year curves presented in the bottom row also enable us to examine whether there were marked increases in concealed carry in 2014 when concealed carry permits began to be issued in Illinois for individuals aged 21 and older. In the cohort curves by year, average cohort 21st birthdays are marked with circles, although no sudden increases occur at these points for any cohorts except the youngest (Fig. 2F). This finding is what we would expect because only the 1996 cohort became eligible for legal carry at the time of their 21st birthday (i.e., when the other cohorts reached 21, it was still illegal). However, no notable increase is seen in the curves for the other cohorts when carry was legalized in Illinois in 2014.

**Fig. 3.**
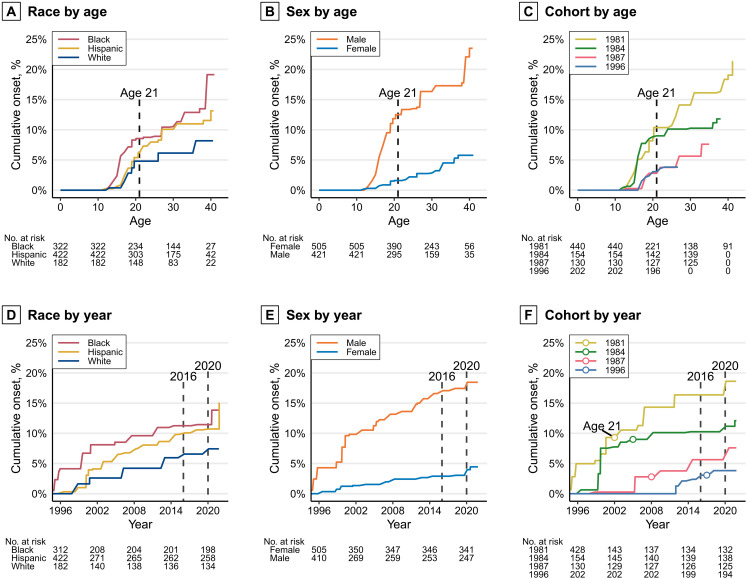
Nonparametric estimates of the cumulative probability of first gun use stratified by race, sex, and birth cohort. (**A** to **C**) Top row: Curves by respondent age. (**D** to **F**) Bottom row: Curves by year. In the cohort by year (F), a circle marks the approximate time when each cohort reached age 21. We estimated these curves of concealed carry by age separately for race, sex, and birth cohort using Turnbull’s (*52*, *53*) NPMLE.

Figure 3 replicates the survival curves above using the age of first reported gun use. These curves largely replicate the patterns seen in Fig. 2: A rapid increase among males in adolescence and then a plateau near age 21; by age 21, the estimated percentage of people who ever shot or shot at someone or used a gun in self-defense was 6.4% (95% CI: 1.4 to 11.1), 7.5% by age 30 (95% CI: 2.4 to 12.4), and 10.1% by age 40 (95% CI: 4.6 to 15.4). Similar to concealed carry, we observe increases in gun use among all older cohorts in 2020. In contrast, there is no notable increase near 2016 (i.e., when Donald J. Trump was elected president and a period following several high-profile police killings of Black individuals).

The discontinuities in the carry curves suggest different processes driving the onset of concealed carry in adolescence and adulthood. Accordingly, the subsequent analyses examine two stages of the life course of concealed carry onset: (i) the rapid increase in adolescence (adolescent onset) and (ii) the gradual but sustained increase through adulthood (adult onset) ending with the recent cross-cutting period-specific increase seen in 2020–2021. We divide these stages at age 21, which is both the typical age of eligibility for legal concealed carry and the end of the plateau of onset in late adolescence observed in the top row of Fig. 2. Our results are unaffected using ages as early as 18 as an alternate age distinction.

### Continuity in carry

**Fig. 4.**
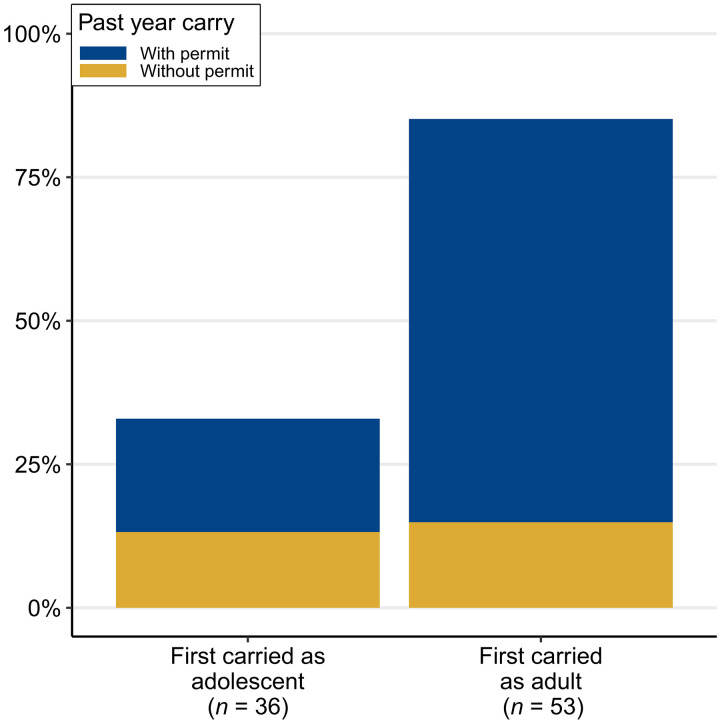
Rates of recent concealed carry (2020–2021) by age when first carried a concealed gun. Percentages were calculated within each onset group (e.g., among the adolescent-onset group) to facilitate comparison.

Figure 4 plots the relationship between the life course stage when individuals first carried a gun (adolescence versus adulthood) and gun carrying in the year before the wave 5 survey conducted in 2021. Percentages were calculated within each group to show how timing of carrying onset is related to recent carrying behavior.

In total, an estimated 13.8% (95% CI: 9.4 to 19.8) of all respondents carried a gun in the past year. As shown in Fig. 4, past-year carry is more than twice as likely for sample members who did not begin carrying a gun until adulthood relative to individuals who began carrying in adolescence: 36.8% (95% CI: 18.6 to 59.8) of adolescent-onset carriers carried (with or without a permit) in the year before survey compared to 85.4% (95% CI: 69.0 to 93.9) of adult-onset carriers. This is driven primarily by differences in permitted carry: 23.0% (95% CI: 8.7 to 48.4) of adolescent-onset carriers versus 69.6% (95% CI: 52.4 to 82.6) of adult-onset carriers carried with a permit in the past year. In contrast, unpermitted carry was similarly likely for adolescent-onset (13.8%, 95% CI: 5.1 to 32.6) and adult-onset carriers (15.9%, 95% CI: 7.2 to 31.3).

Another way to interpret the finding for the group who first carried as an adolescent is that, although all carried a gun illegally as an adolescent (by definition), 62.5% of those who carried a gun in the last year did so legally (23.0%/36.8%). These results suggest that concealed carry that begins in adolescence usually does not translate into life-long persistent carrying, especially life-long illegal carrying, while most concealed carry that begins in adulthood is persistent.

### Gun use by timing of concealed carry

**Fig. 5.**
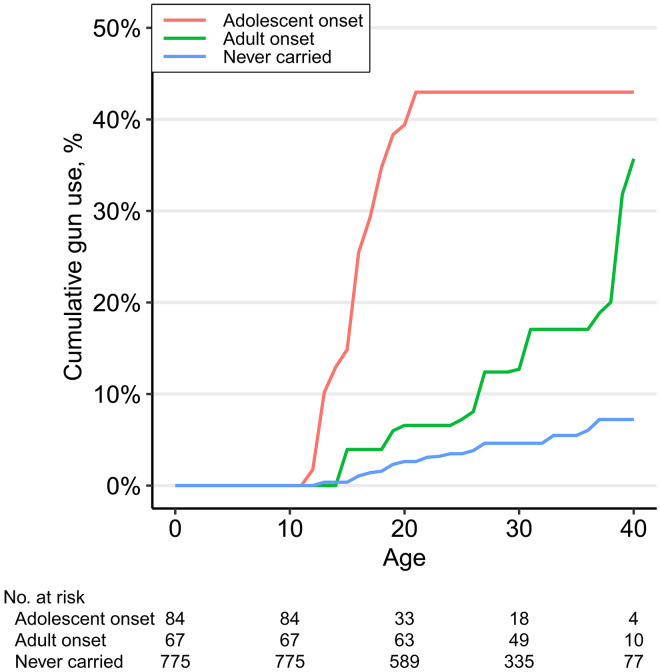
Cumulative percentage of respondents reporting using a gun for defense or shooting at someone by age and timing of onset of concealed carry. No respondents who reported starting to carry a concealed firearm before the age of 21 reported using a gun for the first time after the age of 21.

Figure 5 depicts the percentages of individuals who used a gun within each onset group. The data reveal that gun use is concentrated among individuals who ever carried a concealed weapon, at 26.8% by age 21 (95% CI: 7.4 to 42.1), 30.3% by age 30 (95% CI: 11.0 to 45.5), and 37.4% by age 40 (95% CI: 17.6 to 52.4).

The estimated percentage of people who used a gun by age 40 is greatest among the adolescent-onset gun carriers, but not markedly more than the adult-onset carriers. However, each group takes a much different trajectory to reach this approximately 40% prevalence of gun use by age 40. As seen in Fig. 5, while 43% (95% CI: 27.2 to 55.3) of adolescent-onset gun carriers reported using a gun by age 21, the line remains completely flat after the age of 21, meaning none of the 57% of adolescent-onset carriers who reached age 21 without having used a gun reported ever using a gun later in adulthood; despite their early onset of gun carrying, the observed probability of first gun use drops to zero once they reach adulthood. Because adolescent-onset perfectly predicts the absence of later first gun use, no meaningful statistical analysis can be conducted. In general, however, the onset of concealed carry is a strong predictor of later gun use. As indicated by the “never carried” line in blue, gun use rarely occurs in the absence of concealed carry onset. Half of noncarriers that reported using a gun indicated that their gun use was in response to crimes including burglary, theft, or trespassing.

The adult-onset group follows a starkly different trajectory (green line). Despite adult-onset gun carriers reporting lower risks of gun use year-to-year, by age 40, the estimated lifetime risk of ever using a gun approaches that of the adolescent-onset carriers (35.7%; 95% CI: 8.3 to 54.9). This finding may reflect the high persistence in carry observed in Fig. 4 and period-specific changes in 2020–2021 observed in the bottom row of Fig. 3, although we note that only one of our cohorts had reached age 40 by the end of our observation period.

### Exposure to gun violence

**Fig. 6.**
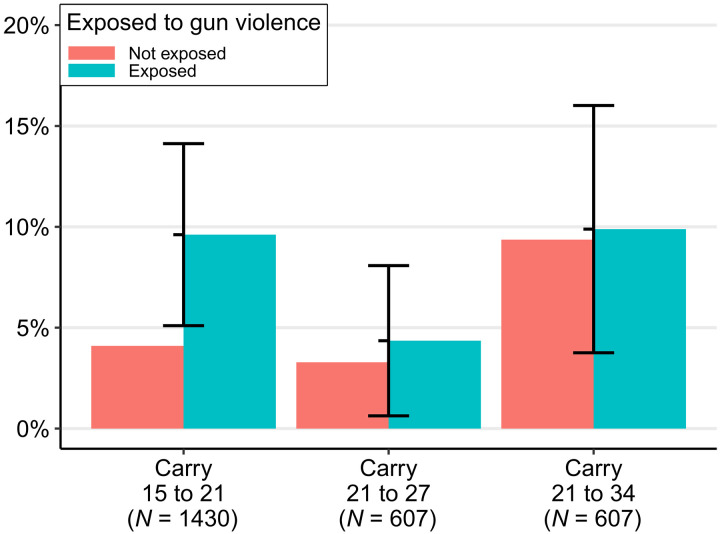
Average predicted probabilities from Firth logit and Cox PH models adjusted for family and neighborhood characteristics. Carry 15 to 21 and 21 to 27 estimated by logistic regression. Carry 21 to 34 estimated by Cox proportional hazard model. Vertical bars are 95% CIs for the difference in probability of carry onset between those exposed and those not exposed. These differences are statistically significant if the interval does not overlap the “not exposed” bar (i.e., for carry 15 to 21).

On the basis of the sharp differences in gun use observed between adolescent and adult-onset groups, we examine next how direct exposure to gun violence through victimization or witnessing shootings relates to onset of concealed carry before and after the age of 21 using multivariable models. Figure 6 displays estimated probabilities from three separate analyses: (i) an adolescent-onset model in which exposure to gun violence before age 15 predicts carry between 15 and 21; (ii) a young adult–onset model in which exposure before age 21 predicts carry between 21 and 27; and (iii) an adult-onset model in which exposure before 21 predicts carry between ages 21 to 34. See fig. S2 for full parameter estimates.

As seen in Fig. 6, exposure to gun violence before age 15 is associated with a statistically significant (*P* < 0.01) doubling in the probability of carrying a concealed gun between ages 15 and 21. In contrast, however, we find exposure to gun violence, through age 21, is not statistically or substantively associated with concealed carry onset between 21 and 27 or 21 to 34. As the results for carry 21 to 34 reveal, 10% of individuals who reached 21 without carrying a concealed gun are expected to carry one by age 34, whether exposed to violence or not. Direct exposure to gun violence after the age of 21 is far less frequent than during adolescence, precluding estimates of the effect of adult exposure on adult gun carrying, but we would suggest that the modest adult exposure to violence likely has a minimal effect on the relatively pervasive gun carrying in adulthood. Hence, as contemplated in the Discussion, it appears that concealed carrying of guns in late adolescence is, at least in part, the product of proximal circumstances and situations such as violent environments, but concealed carrying is mainly the product of other forces in adulthood.

While we have insufficient statistical power to estimate interaction effects, it appears that the difference in the probability of carrying a concealed weapon between exposed and unexposed respondents (regardless of when in the life course) is largest for white respondents (almost 10 times higher) compared to Black (2.5 times higher) or Hispanic (0.5 times higher) respondents. This is consistent with those least likely to be exposed to gun violence (i.e., white respondents) being the most likely to begin carrying a firearm in reaction to being exposed.

## DISCUSSION

This paper contributes to a burgeoning literature on gun violence by examining concealed gun carrying, and its association with exposure to gun violence, well into adulthood. Previously, much of the extant literature has been narrowly focused on adolescence. As a result, prior research has been unable to sufficiently demonstrate the dynamic nature of gun-related behavior, including questions about its continuity from adolescence into adulthood. A contribution of our paper has been to bring a life course perspective to the study of concealed carry and use, which conceptually allows for the possibility that gun behavior and the underlying attitudes and situations that drive this behavior change over time, sometimes quite markedly. Before summarizing and discussing our findings, we highlight several limitations that present opportunities for future research.

The PHDCN+ data collection is based on following cohorts originally from Chicago, raising the question of generalizability. While our findings help contextualize broader trends related to gun carrying and its correlates, further research in broader settings is necessary to confirm the applicability of our findings to the national context.

Although the PHDCN+ collected granular data on the timing of first occurrences of gun carry, gun use, and exposure to gun violence over a 25-year period in respondents’ lives, it did not collect data on the frequency of these events. More frequent data collection would be required to obtain accurate data on frequency of carry, but these data would permit determining whether the relationship between exposure to gun violence and carrying is stronger for those with multiple exposures or whether adolescent-onset carriers who reported gun use in adolescence also used guns during adulthood.

Relatedly, we are only able to measure continuity in carry using past year carry. If sharp increases in gun purchases nationwide in 2020–2021 were accompanied by similar increases in gun carrying among our respondents, then the past year under consideration here may be one with unusually high rates of carry. If so, typical rates of continuity may be lower than observed. Results from nationwide surveys on gun carrying may be suggestive (*18*). We also observe a modest amount of nonresponse to gun carrying and use questions as shown in fig S1. However, nearly all nonresponse is limited to adolescent respondents who were not observed after wave 3; only four respondents of wave 5 declined to answer. Accordingly, while nonresponse bias may affect prevalence estimates in adolescence (e.g., underestimating carry if carriers are reluctant to report), nonresponse is too low to affect our key findings on adult onset and continuity, which are derived from wave 5 data (*N* = 4 nonresponses).

Despite these limitations, our analyses reveal several findings that bear implications for future research and policy. One simple but crucial fact concerns prevalence: Carrying a concealed firearm is now a common event in the life course, with an estimated 31.9% (95% CI: 17.9 to 43.6) of our study population carrying by age 40. This pattern, however, differs widely by race, sex, and birth timing. By age 40, an estimated 47.6% (95% CI: 26.0 to 62.8) of males had carried a concealed gun compared to only 16.0% (95% CI: 6.7 to 24.4) of females. Racial differences persisted over the life course with a convergence of white and Hispanic respondents by age 40 and rates over two times as great for Black respondents. These patterns also differed by birth cohort, with continually higher rates for those born in 1981, but a convergence of the 1987 cohort to the 1981 rates and much lower rates of carrying concealed firearms among the cohorts born in 1984 and 1996.

Many of the increases in carrying that we observed in the descriptive plots over time can be attributed to period effects from sociohistorical events in 2016 and 2020, which resulted in spikes in violence nationwide. For example, although a small number of overall respondents (3%), 24% of respondents who reported carrying in the past year in 2021 were new to carrying. We believe that this is an important group that would be overlooked in research focused on adolescent- or young adult–onset carrying. Moreover, while the limited longitudinal evidence on adult carrying suggests that handgun carrying was stable year to year during the first decade of the 2000s (*19*), the fact that nearly one-quarter of recent gun carriers in our data are in fact new carriers suggests that this stability may have been disrupted by the macro-historical events such as COVID that have reshaped contemporary society.

We also find that life course onset and continuity in carrying—and related gun use—follow two contrasting patterns, or what we term a dual pathway model, that has not previously been evident, likely because of the paucity of long-term data on gun carrying and use. First, adolescent-onset carriers with first carry before the age of 21 account for one-third of all lifetime concealed gun carriers. This carrying was initially illegal, and the carrying and gun use are usually age-limited; most of these individuals have not carried recently. Whereas 43% of the adolescent-onset carriers started using a gun (shooting or brandishing) while still in adolescence (and may have continued to use guns as adults), the other 57% never engaged in gun use, whether as adolescents or as adults. Personal exposure to gun violence is a strong predictor of adolescent onset of carry. This evidence adds to our understanding of adolescent gun offending (*38*, *39*), but future research should explore the mechanisms leading to the discontinuation of gun carrying. Moving out of high violence areas or aging into periods with relatively less violence may explain these patterns.

Second, adult-onset carriers who first carry after the age of 21 account for a substantial two-thirds of all lifetime carriers. Their carry is more likely persistent in the sense that they also reported carrying recently, and moreover, their gun use accumulates over time; the great majority have carried recently, and they exhibit a steady increase in onset of gun use with age after having very low rates of gun use in adolescence. In contrast to adolescent-onset of carrying, prior exposure to gun violence, is not significantly predictive of adult-onset carry.

Whereas there are marked differences between the two groups, there are also similarities, as we show in table S2. The adolescent-onset carriers are, by definition, individuals engaged in criminal conduct (i.e., gun crimes), and the majority of them self-reported getting arrested (78%) at some point in their lives, almost all of whom (95%) were arrested for the first time in adolescence. Yet, a majority (61%) of the adult-onset group also have a history of arrest, with more than half of those with an arrest record first getting arrested as an adolescent (51%). In contrast, only 31% of individuals who never carried a gun report an arrest record, a percentage that is consistent with national estimates (*40*).

A natural question arises from these findings: What causes selection into each distinct pathway, adolescent versus adult carrying of concealed guns, particularly since, as noted above, both groups displayed a high degree of criminality during adolescence? One explanation is that gun carrying in adolescence is illegal, whereas gun carrying in adulthood was potentially legal for most adults in our sample after gun carrying laws became more permissive in Illinois and nationwide. Hence, criminological explanations for the relatively steep decline in criminal offending in early adulthood may be relevant for explaining why the adolescent gun carriers in our sample desisted from carrying (*38*, *41*). For instance, the adolescent-limited group may have stopped carrying when they reached the end of adolescence because they obtained gainful employment or established family bonds that provided greater controls on behavior. Of relevance, gun carrying is not a behavior that is uniformly antisocial across the life course but rather one that is, similar to alcohol use, age-graded in legality and normative acceptability. In contrast to gun carrying, however, adolescent alcohol consumption is strongly predictive of drinking over the life course, particularly problem drinking (*42*).

A critical distinction between the adolescent-onset versus adult-onset gun concealed carry groups is the role of the immediate social context of violence. Past research on adolescent gun carry and our own results suggest that immediately dangerous contexts—such as those marked by direct exposure to gun violence—lead to adolescent gun carrying (*12*, *25*, *28*). In contrast, we find no association between prior exposure to gun violence and gun carrying for adults, but we do find a sharp increase in adult gun carrying in 2020–2021 that coincides with increases in gun purchases seen throughout the United States following the outbreak of COVID-19 and murder of George Floyd (*6*, *7*).

These results evoke Stroebe *et al.*’s (*43*) findings that protective gun ownership among adult men is associated primarily with diffuse threats—e.g., “the belief that the world is a dangerous and unstable place, populated by bad people, and that society is at the brink of collapse” [page 1079 in (*43*)]—and secondarily by specific threats—e.g., perceived likelihood of victimization (*44*). This is reflected in a recent survey that finds gun owners—and particularly regular gun carriers—are more likely to consider violence justified to “reinforce police,” resist oppressive government, and protect the “American Way of Life,” as well as to believe a civil war is imminent (*45*).

In an argument that evokes the broader literature on legal cynicism (*30*, *46*), Carlson (*10*, *47*) finds that two racially differentiated beliefs promote legal gun carrying: The belief common among most carriers that police are inadequate protectors—and thus one may carry a gun as protection from crime—and the belief more common among non-white carriers that police are coercive violators of rights—and thus one may carry a gun as protection from and resistance to the oppressive state (*48*). In 2020–2021, social unrest and economic disruption as well as the resulting perceptions of lawlessness (*33*), spikes in gun violence (*15*), and a crisis of police legitimacy (*49*) likely acted on all of these mechanisms at once (*50*). In broader terms, all members of our sample—and Americans of similar age—grew up in a period of self-defense oriented American gun culture (*48*). Accordingly, although our analyses are focused on birth cohorts originally from Chicago, these macro-historical events and the feelings of uncertainty and insecurity that they sparked are not unique to the city, and we suggest that they influence gun carrying throughout the United States.

These findings suggest that changing macro-historical contexts affect the frequency and timing of concealed carry over the life course of individuals, such as by increasing their perceived needs for protection. This in turn raises a new question: How does variation in individual gun carrying affect macro-historical context such as aggregate rates of gun violence in the United States? The nation has seen large swings in lethal gun violence over the last four decades, and we similarly observe large swings in time-specific onset of concealed carry, most recently in 2020. To what extent are these swings related to changes in concealed carry? This is a difficult question to answer without national longitudinal data on concealed carry, although recent research suggests that the shift from may-issue to shall-issue concealed carry laws has had a sizable impact on firearm violence (*3*). While flawed, state-level permit records are one of the few resources available for tracking carry prevalence, but the rapid expansion of permitless carry will eliminate even this measurement tool. As a result, survey data—particularly longitudinal survey data—will become increasingly important for understanding gun carrying behavior and its consequences.

## MATERIALS AND METHODS

We preregistered the research design for this study on Open Science Framework (*51*), with the only deviation from the preregistration being our decision to double the number of imputed datasets from 25 to 50 in the exposure to gun violence analyses to minimize variation in the final estimates. We estimated curves of the cumulative proportion of respondents reporting onset of concealed carry by age separately for race, sex, and birth cohort in Fig. 2 using Turnbull’s (*52*, *53*) nonparametric maximum-likelihood estimator (NPMLE). This estimator is analogous to the common Kaplan-Meier estimator but is applicable to interval-censored data. This is necessary because the onset of concealed carry is reported as having occurred within an interval such as at a particular age; this means that onset could have occurred at any point within the year-long interval that the respondent was that age. Estimates from the survival curves are descriptions of the life-course timing of the onset of concealed carry rather than causal analyses. For example, being a member of a particular birth cohort does not directly cause differences in concealed carry, but rather differentiation across birth cohorts may be driven by exposure to different historical contexts. An advantage of the accelerated cohort design of the PHDCN+ is that it allows for distinguishing between age-specific and period-specific effects because each cohort reaches a particular age at different times (*13*). Cumulative proportions of respondents reporting onset of gun use (Figs. 3 and 5) were also calculated using the NPMLE but should be interpreted with caution due to the relative rarity of events (*n* = 75 for gun use compared to *n* = 199 for carry).

For all NPMLE curves, diagonal segments indicate regions where the curves are not uniquely identified due to interval censoring; any monotonically increasing line connecting the ends of diagonal regions is consistent with the observed data. Curves for each stratum were truncated once the number of respondents at-risk drops below 10. Note that due to the combination of mixed (i.e., interval and right) censoring, the at-risk sample size varies over time, and thus, the estimated cumulative prevalences do not correspond directly to the total number of observed events divided by the sample size. Estimated at-risk sample sizes are displayed at the bottom of the figures.

Continuity in carry (Fig. 4)—i.e., carrying in the past and in the year leading up to our most recent wave of data collection—was examined using survey-weighted frequencies of past-year gun carrying among the 642 Black, Hispanic, and white respondents of the most recent survey administered in 2021. As focus is on continuity, we separate respondents who first carried within 1 year of the survey (i.e., new adult carriers) from other adult-onset carriers who started carrying more than 1 year ago. Weighted onset group sample sizes are displayed in the bottom margin of Fig. 4.

Associations between exposure to gun violence and gun carrying (Fig. 6 and fig. S2) were estimated using a counterfactual approach in which exposure to gun violence before a given age $T_{1}$ is used as a predictor of carry onset in the interval between age $T_{1}$ and an end-point age $T_{2}$. Respondents who reported onset of carry before the period under consideration are excluded. For example, in the first model, $T_{1}=15$ and $T_{2}=21$, meaning that exposure to gun violence before the age of 15 is used to predict carry onset occurring between age 15 and 21, and respondents who reported onset of carry before the age of 15 are excluded from the analysis. This approach ensures that exposure is clearly defined and temporally precedes onset of carry. Three separate analyses were conducted: (i) the aforementioned adolescent-onset model in which exposure before the age of 15 predicts carry between 15 and 21; (ii) a young adult–onset model in which exposure before the age of 21 predicts carry between 21 and 27; and (iii) an adult-onset model in which exposure before 21 predicts carry after the age of 21 to 34.

The adolescent-onset range of 15 to 21 was chosen because adolescent-onset carry is highly concentrated in ages 15 to 18, but the average age when exposed to gun violence in this sample is 14 (*14*). Results are substantively identical using different values of *T*_1_ (e.g., 14 or 16). The first adult-onset range of 21 to 27 was chosen to yield an interval of equal length to the adolescent interval for comparison and to minimize bias from right censoring among the 1996 cohort who were, on average, 26 at survey time. Results are substantively identical using different values of *T*_1_ (e.g., 18 to 20) or *T*_2_ (e.g., 25 or 26). The second adult-onset model uses 34 as an end point because nearly all members of the oldest three cohorts had reached this age by the time of the wave 5 survey. Exposure during adulthood, e.g., between 21 and 27, was too infrequent to estimate models.

The first two models presented in Fig. 6 were estimated using logistic regression with a bias correction for rare outcomes (*54*). The third model was estimated using a Cox proportional hazards model to address right censoring. A weighted residual test indicates that the proportional hazard assumption is not satisfied between cohorts. We present the Cox model estimates because the substantive interpretation is identical when using parametric accelerated failure time and proportional odds models. Estimates in all three models are adjusted for the same battery of covariates including the core strata variables seen in the cumulative onset curves (race, sex, and age cohort), as well as childhood socioeconomic status (SES), immigrant generation of the respondent’s primary caregiver (immigrant family), history of arrest in the family (family arrest), and four census tract measures: the 3-year moving average of the homicide rate (*55*, *56*), percent Black population, population density, and percent of households living under the poverty line (*57*).

Concealed carry and exposure to violence are interval censored measures, but exact times are required for estimation; these were imputed using the semi-parametric Turnbull proportional hazards model that estimates the baseline hazard using the NPMLE (*52*, *58*). Because of the computational demands of the NPMLE, this could not be performed at the same time as covariate imputation for missing data. Instead, covariates were first imputed in 50 datasets using chained equations with 20 iterations. Sociodemographic variables had very few missing values. No values were missing for the race, sex, or cohort. Thirty-eight (1.8%) values of SES, 30 values of family arrest (1.4%), and 25 (1.2%) values of caregiver immigrant generation were imputed. In contrast, missingness is higher for neighborhood variables: 274 (12.9%) of cases in the carry 15 to 21 models were missing age 12 measures, and 148 (22.9%) of cases in the carry 21 to 27 and 21 to 34 models were missing age 18 measures. This is typically due to the absence of homicide measures for those living outside the city of Chicago. After covariates were imputed, the exact times of onset were singly imputed for each of the 50 datasets. Imputation was conducted by first drawing a random sample of coefficients under the assumption of asymptotic normality, then predicting the exact response time conditional on the observed covariates and censoring interval (*52*). The exposure models were then estimated separately on each of the 50 datasets, and the results were pooled using Rubin’s rules (*59*). Variation in imputed exposure and carry onset times produces small variations in model sample sizes. For example, a case could be dropped from the 15 to 21 onset model if the imputed age of onset is younger than 15. This results in sample sizes that vary from 1423 to 1439 for exposure 15 to 21 and from 607 to 609 for exposure 21 to 27 and 27 to 34. Median sample sizes are reported in Fig. 5. Results are indistinguishable when excluding the neighborhood covariates or using listwise deletion on the small number of cases with missing individual-level covariates. Full parameter estimates from these models are displayed in fig. S3.

Estimates in all analyses were weighted for features of the survey design and attrition to permit making inferences back to the population of Chicago’s children in the mid-1990s from which the sample was drawn. The survey design weights account for the stratified random sampling design at baseline. Attrition weights were estimated using logistic regression with a broad set of individual, family, and neighborhood covariates, including the following: the respondent’s sex, race/ethnicity, cohort, age at last survey, and number of arrests in official records; their primary caregiver’s sex, immigrant generation, age, household SES, household size, marital status, and social ties; and characteristics of the respondent’s neighborhood during the prior wave, including racial and socioeconomic composition [see also page 523 in (*34*)]. Attrition weights for each wave were calculated by taking the inverse of each subject’s probability of response and standardizing these values by dividing by the mean. To produce the final analysis weights, the design and attrition weights were then combined and trimmed to the central 90% to reduce the influence of extreme weights. The interpretations of our results (e.g., proportional inequalities) are unchanged when using unweighted estimates or limiting our analyses only to the 642 respondents from wave 5.

## References

[1] J. Kaplan, Jacob Kaplan’s concatenated files: Uniform Crime Reporting (UCR) program data: Supplementary Homicide Reports (SHR), 1976–2021, version v12 (Interuniversity Consortium for Political and Social Research, 2023); 10.3886/E100699V12.

[2] S. N. Oliphant, C. A. Mouch, A. Rowhani-Rahbar, S. Hargarten, J. Jay, D. Hemenway, M. Zimmerman, P. M. Carter; for the FACTS Consortium, A scoping review of patterns, motives, and risk and protective factors for adolescent firearm carriage. J. Behav. Med. 42, 763–810 (2019). 31367939 10.1007/s10865-019-00048-x PMC7182091

[3] M. L. Doucette, A. D. McCourt, C. K. Crifasi, D. W. Webster, Impact of changes to concealed-carry weapons laws on fatal and nonfatal violent crime, 1980–2019. Am. J. Epidemiol. 192, 342–355 (2023). 36104849 10.1093/aje/kwac160

[4] J. Mascia, C. Brownlee, “How many guns are circulating in the US?,” *The Trace*, 6 March 2023; www.thetrace.org/2023/03/guns-america-data-atf-total/.

[5] B. J. Lang, M. Lang, Pandemics, protests, and firearms. Am. J. Health Econ. 7, 131–163 (2021).

[6] M. Miller, W. Zhang, D. Azrael, Firearm purchasing during the COVID-19 pandemic: Results from the 2021 National Firearms Survey. Ann. Intern. Med. 175, 219–225 (2022). 34928699 10.7326/M21-3423 PMC8697522

[7] Federal Bureau of Investigation, “NICS Firearm Checks: Month/Year” (2023); www.fbi.gov/file-repository/nics_firearm_checks_-_month_year.pdf/view.

[8] U.S. Bureau of Alcohol, Tobacco, Firearms, and Explosives, “National Firearms Commerce and Trafficking Assessment (NFCTA): Crime Guns - Volume Two” (2023); www.atf.gov/firearms/national-firearms-commerce-and-trafficking-assessment-nfcta-crime-guns-volume-two.

[9] G. H. Elder Jr., Age differentiation and the life course. Annu. Rev. Sociol. 1, 165–190 (1975).

[10] J. Carlson, *Citizen-Protectors: The Everyday Politics of Guns in an Age of Decline* (Oxford Univ. Press, 2015).

[11] A. Stroud, *Good Guys with Guns: The Appeal and Consequences of Concealed Carry* (University of North Carolina Press, 2016).

[12] B. E. Molnar, M. J. Miller, D. Azrael, S. L. Buka, Neighborhood predictors of concealed firearm carrying among children and adolescents: Results from the Project on Human Development in Chicago Neighborhoods. Arch. Pediatr. Adolesc. Med. 158, 657–664 (2004). 15237065 10.1001/archpedi.158.7.657

[13] R. Neil, R. J. Sampson, The birth lottery of history: Arrest over the life course of multiple cohorts coming of age, 1995–2018. Am. J. Sociol. 126, 1127–1178 (2021).

[14] C. C. Lanfear, R. Bucci, D. S. Kirk, R. J. Sampson, Inequalities in exposure to firearm violence by race, sex, and birth cohort from childhood to age 40 years, 1995-2021. JAMA Netw. Open 6, e2312465 (2023). 37159198 10.1001/jamanetworkopen.2023.12465 PMC10170342

[15] R. Rosenfeld, B. Boxerman, E. Lopez, “Pandemic, social unrest, and crime in US cities: Year-end 2022 Update” (Council on Criminal Justice, 2023).

[16] C. Brownlee, “Permitless carry: These states allow gun owners to carry without a license,” *The Trace*, 12 May 2023; www.thetrace.org/2023/05/permitless-carry-gun-laws-states-map/.

[17] K. Parker, J. M. Horowitz, R. Igielnik, J. B. Oliphant, A. Brown, “America’s complex relationship with guns” (Pew Research Center, 2017); https://policycommons.net/artifacts/617827/americas-complex-relationship-with-guns/1598689/.

[18] A. Rowhani-Rahbar, A. Gallagher, D. Azrael, M. Miller, Trend in loaded handgun carrying among adult handgun owners in the United States, 2015–2019. Am. J. Public Health 112, 1783–1790 (2022). 36383941 10.2105/AJPH.2022.307094 PMC9670230

[19] B. P. Comer, E. J. Connolly, Exposure to gun violence and handgun carrying from adolescence to adulthood. Soc. Sci. Med. 328, 115984 (2023). 37245260 10.1016/j.socscimed.2023.115984

[20] K. Schaeffer, “Key facts about Americans and guns” (Pew Research Center, 2023); www.pewresearch.org/short-reads/2023/09/13/key-facts-about-americans-and-guns/.

[21] J. F. Sheley, J. D. Wright, “Gun acquisition and possession in selected juvenile samples” (145326, US Department of Justice, Office of Justice Programs, National Institute of Justice, 1993); www.ojp.gov/library/publications/gun-acquisition-and-possession-selected-juvenile-samples.

[22] H. Shapira, C. Liang, K.-H. Lin, How attitudes about guns develop over time. Sociol. Perspect. 65, 12–34 (2022).

[23] A. Blumstein, Youth violence, guns, and the illicit-drug industry. J. Crim. Law Criminol. 86, 10–36 (1995).

[24] A. J. Lizotte, M. D. Krohn, J. C. Howell, K. Tobin, G. J. Howard, Factors influencing gun carrying among young urban males over the adolescent-young adult life course. Criminology 38, 811–834 (2000).

[25] J. B. Bingenheimer, R. T. Brennan, F. J. Earls, Firearm violence exposure and serious violent behavior. Science 308, 1323–1326 (2005). 15919997 10.1126/science.1110096

[26] R. Spano, J. M. Bolland, Is the nexus of gang membership, exposure to violence, and violent behavior a key determinant of first time gun carrying for urban minority youth. Justice Q. 28, 838–862 (2011).

[27] L. A. Teplin, N. S. Meyerson, J. A. Jakubowski, D. A. Aaby, N. Zheng, K. M. Abram, L. J. Welty, Association of firearm access, use, and victimization during adolescence with firearm perpetration during adulthood in a 16-year longitudinal study of youth involved in the juvenile justice system. JAMA Netw. Open 4, e2034208 (2021). 33538822 10.1001/jamanetworkopen.2020.34208 PMC7862991

[28] J. Beardslee, E. Mulvey, C. Schubert, P. Allison, A. Infante, D. Pardini, Gun- and non-gun–related violence exposure and risk for subsequent gun carrying among male juvenile offenders. J. Am. Acad. Child Adolesc. Psychiatry 57, 274–279 (2018). 29588053 10.1016/j.jaac.2018.01.012 PMC5876872

[29] H. Shapira, K. Jensen, K.-H. Lin, Trends and patterns of concealed handgun license applications: A multistate analysis. Soc. Curr. 5, 3–14 (2018). 38881886 10.1177/2329496517725334 PMC11177889

[30] D. S. Kirk, A. V. Papachristos, Cultural mechanisms and the persistence of neighborhood violence. Am. J. Sociol. 116, 1190–1233 (2011). 10.1086/655754 21648250

[31] E. Depetris-Chauvin, Fear of Obama: An empirical study of the demand for guns and the U.S. 2008 presidential election. J. Public Econ. 130, 66–79 (2015).

[32] P. Levine, R. McKnight, “Three million more guns” (Brookings Institution, 2020); https://policycommons.net/artifacts/4139054/three-million-more-guns/4947646/.

[33] N. Kravitz-Wirtz, A. Aubel, J. Schleimer, R. Pallin, G. Wintemute, Public concern about violence, firearms, and the COVID-19 pandemic in California. JAMA Netw. Open 4, e2033484 (2021). 33394004 10.1001/jamanetworkopen.2020.33484 PMC7783542

[34] R. J. Sampson, D. S. Kirk, R. Bucci, Cohort profile: Project on human development in Chicago neighborhoods and its additions (PHDCN+). J. Dev. Life-Course Criminol. 8, 516–532 (2022). 35669615 10.1007/s40865-022-00203-0 PMC9156356

[35] K. M. Harris, C. T. Halpern, E. A. Whitsel, J. M. Hussey, L. A. Killeya-Jones, J. Tabor, S. C. Dean, The national longitudinal study of adolescent to adult health (Add Health): An underused resource for developmental science. Annu. Rev. Dev. Psychol. 4, 297–318 (2022).

[36] P. J. Cook, J. Ludwig, “Guns in America: National survey on private ownership and use of firearms” (National Institute of Justice, 1997).

[37] B. M. Hicks, C. Vitro, E. Johnson, C. Sherman, M. M. Heitzeg, C. E. Durbin, E. Verona, Who bought a gun during the COVID-19 pandemic in the United States?: Associations with QAnon beliefs, right-wing political attitudes, intimate partner violence, antisocial behavior, suicidality, and mental health and substance use problems. PLOS ONE 18, e0290770 (2023). 37643192 10.1371/journal.pone.0290770 PMC10464976

[38] T. E. Moffitt, Adolescence-limited and life-course-persistent antisocial behavior: A developmental taxonomy. Psychol. Rev. 100, 674–701 (1993). 8255953

[39] T. E. Moffitt, Male antisocial behaviour in adolescence and beyond. Nat. Hum. Behav. 2, 177–186 (2018). 30271880 PMC6157602

[40] U.S. Department of Justice, “Survey of state criminal history information systems, 2012” (Bureau of Justice Statistics, 2014; www.ncjrs.gov/pdffiles1/bjs/grants/244563.pdf.

[41] R. J. Sampson, J. H. Laub, *Crime in the Making: Pathways and Turning Points through Life* (Harvard Univ. Press, 1995).

[42] Á. Szabó, A. Towers, D. Newcombe, J. Sheridan, Alcohol use trajectories across the life course: Influences of childhood predictors and consequences for late-life health. Drug Alcohol Depend. 224, 108713 (2021). 33940326 10.1016/j.drugalcdep.2021.108713

[43] W. Stroebe, N. P. Leander, A. W. Kruglanski, Is it a dangerous world out there? The motivational bases of American gun ownership. Pers. Soc. Psychol. Bull. 43, 1071–1085 (2017). 28903713 10.1177/0146167217703952

[44] T. D. Warner, C. R. Thrash, A matter of degree? Fear, anxiety, and protective gun ownership in the United States. Soc. Sci. Q. 101, 285–308 (2020).

[45] G. J. Wintemute, A. Crawford, S. L. Robinson, E. A. Tomsich, P. M. Reeping, J. P. Schleimer, V. A. Pear, Firearm ownership and support for political violence in the United States. JAMA Netw. Open 7, e243623 (2024). 38592725 10.1001/jamanetworkopen.2024.3623 PMC11004826

[46] R. J. Sampson, D. J. Bartusch, Legal cynicism and (subcultural?) tolerance of deviance: The neighborhood context of racial differences. Law Soc. Rev. 32, 777–804 (1998).

[47] J. D. Carlson, “I don’t dial 911”: American gun politics and the problem of policing. Br. J. Criminol. 52, 1113–1132 (2012).

[48] D. Yamane, Gun culture 2.0: The evolution and contours of defensive gun ownership in America. Ann. Am. Acad. Pol. Soc. Sci. 704, 20–43 (2022).

[49] T. T. Reny, B. J. Newman, The opinion-mobilizing effect of social protest against police violence: Evidence from the 2020 George Floyd protests. Am. Polit. Sci. Rev. 115, 1499–1507 (2021).

[50] R. J. Sampson, C. C. Lanfear, Disentangling gun ownership and leanings to political violence in unstable times. JAMA Netw. Open 7, e245066 (2024). 38592726 10.1001/jamanetworkopen.2024.5066

[51] D. Kirk, C. C. Lanfear, R. Sampson, R. Bucci, Gun Carrying from Adolescence to Adulthood: Initiation, Continuity, and Exposure to Gun Violence Across the Life Course. OSF (2024). https://doi:10.17605/OSF.IO/3DBXW.

[52] C. Anderson-Bergman, icenReg: Regression models for interval censored data in R. J. Stat. Softw. 81, 1–23 (2017).

[53] B. W. Turnbull, The empirical distribution function with arbitrarily grouped, censored and truncated data. J. R. Stat. Soc. Series B Stat. Methodol. 38, 290–295 (1976).

[54] I. Kosmidis, E. C. Kenne Pagui, N. Sartori, Mean and median bias reduction in generalized linear models. Stat. Comput. 30, 43–59 (2020).

[55] C. R. Block, R. L. Block, Illinois Criminal Justice Information Authority, Homicides in Chicago, 1965–1995 (Inter-university Consortium for Political and Social Research [distributor] (2005); 10.3886/ICPSR06399.v5.

[56] Chicago Police Department, “Crimes - 2001 to Present” (2020); https://data.cityofchicago.org/Public-Safety/Crimes-2001-to-Present/ijzp-q8t2.

[57] J. R. Logan, Z. Xu, B. J. Stults, Interpolating U.S. decennial census tract data from as early as 1970 to 2010: A longitudinal tract database. Prof. Geogr. 66, 412–420 (2014). 25140068 10.1080/00330124.2014.905156 PMC4134912

[58] C.-H. Hsu, J. M. G. Taylor, S. Murray, D. Commenges, Multiple imputation for interval censored data with auxiliary variables. Stat. Med. 26, 769–781 (2007). 16755528 10.1002/sim.2581

[59] D. B. Rubin, *Multiple Imputation for Nonresponse in Surveys* (Wiley, ed. 1, 1987).

