# Supplementary material for: Dual pathways of concealed gun carrying and use from adolescence to adulthood over a 25-year era of change
Source: Sci Adv. 2024 Dec 4;10(49):eadp8915. doi: 10.1126/sciadv.adp8915 (PMC11616699; doi:10.1126/sciadv.adp8915)
Supplement: Supplementary file 1 — Supplementary Text Figs. S1 to S3 Tables S1 and S2 [file sciadv.adp8915_sm.pdf]

Science Advances
AAAS

Supplementary Materials for

Dual pathways of concealed gun carrying and use from adolescence to adulthood over a 25-year era of change

Charles C. Lanfear *et al.*

Corresponding author: Charles C. Lanfear, cl948@cam.ac.uk

Sci. Adv. **10**, eadp8915 (2024)
DOI: 10.1126/sciadv.adp8915

This PDF file includes:

Supplementary Text
Figs. S1 to S3
Tables S1 and S2

Supplementary Text

Variables used in analyses

All dependent variables are calculated from multiple survey items asked directly to respondents of the PHDCN-LCS. The survey waves and cohorts asked for each item are coded for brevity. For example, "W2,W3:9,12,15" indicates an item asked of respondents in waves 2 and 3 if they were in cohorts 9, 12, and 15. "All" indicates cohorts 0, 9, 12, and 15.

Outcomes

1. Age when respondent first carried a concealed gun (i.e., onset):
    1. "Have you ever carried a concealed or hidden gun with you anywhere?" (W2,W3:9,12,15)
    2. "When was the last time you carried a concealed gun?" (W2,W3:9,12,15; W5:all)
    3. "Have you ever carried a concealed or hidden gun with you anywhere, whether you had a permit for the gun or not? This doesn't include a BB gun or any type of toy gun, like a paint ball gun or air rifle." (W5:all)
    4. "What age were you the first time you carried a concealed or hidden gun with you anywhere?" (W5:all)
2. Past-year concealed gun carry:
    1. "Have you ever carried a concealed or hidden gun with you anywhere, whether you had a permit for the gun or not? This doesn't include a BB gun or any type of toy gun, like a paint ball gun or air rifle?" (W5:all)
    2. "When was the last time you carried a concealed gun?" (W5:all)
    3. "Did you have a concealed carry gun permit the last time you carried a concealed or hidden gun?" (W5:all)
3. Age when respondent first used a gun:
    1. "Have you ever used a gun in self defense?" (W3:15)
    2. "Did this [using a gun in self defense] happen in the past year?" (W3:15)
    3. "Have you ever used a gun, even if it was not fired, to protect yourself, someone else, or your property?" (W5:all)
    4. "How old were you when this [using a gun to protect yourself, someone else or your property] first happened?" (W5:all)
    5. "In your whole life, have you ever shot or shot at somebody?" (W5:all)
    6. "How old were you when this [having shot or shot at somebody] first happened?" (W5:all)

Predictors

1. Age when respondent was first exposed to gun violence:
    1. "Have you ever seen or been present when someone was shot?" (W1:9,12,15)
    2. "When was the last time you saw that [someone shot]?" (W1:9,12,15)
    3. "In your whole life, have you ever seen someone else get shot?" (W2,W3:9,12,15; W5:all)
    4. "Now just thinking about the last 12 months, have you seen someone get shot?" (W2,W3:9,12,15)
    5. "How old were you the first time you saw this [someone get shot]?" (W2:W9,12,15)
    6. "Now just thinking about the past 12 months, have you ever seen someone else get shot?" (W5:all)
    7. "In your whole life, have you ever been shot?" (W2,W3:9,12,15; W5:all)

    8. "About how old were you the first time this [being shot] happened?" (W2,W3:9,12,15)
    9. "Now just thinking about the last 12 months, have you been shot?" (W2,W3:9,12,15)
    10. "How old were you when this [being shot] first happened?" (W5:all)
2. Demographic characteristics of respondents:
    1. Age, based on date of birth and survey date
    2. Sex, as recorded at wave 1 or wave 2 for members of the 1996 cohort that were not yet born at wave 1 of data collection
    3. Race / Ethnicity, as reported by primary caregiver at wave 1
    4. Foreign-born primary caregiver, as reported in wave 1
    5. Family history of arrest or trouble with the police, as reported by primary caregiver at wave 1.
    6. Socioeconomic status (SES) of primary caregiver during respondent's childhood, from waves 1, 2, and 3, is measured using the first principal component of six indicators. To best capture conditions relevant to child development, indicators at wave 3 are used for the 1996 cohort and wave 1 for the other cohorts.
        1. Welfare receipt, i.e., on Temporary Assistance to Needy Families (binary)
        2. Home ownership (binary)
        3. Highest degree attained, ordinal with three levels: (1) Less than high school; (2) high school or GED; (3) bachelor's degree or higher
        4. Household income, ordinal with seven levels: (1) less than $5,000; (2) $5,000-9,999; (3) $10,000-19,999; (4) 20,000-29,999; (5) 30,000-39,999; (6) $40,000-49,999; (7) greater than 50,000
        5. Employment status, i.e., employed or not (binary)
3. Neighborhood context is operationalized with four variables based on official data describing the census tract of residence at a given age 12 or 18. Tract of residence at each age is based on residential history records collected in every survey wave.
    1. Percent of households under poverty line, from Logan et al.'s (*57*) Longitudinal Tract Data Base (LTDB) which compiles data from US Census and American Community Survey (*56*).
    2. Population percent non-Hispanic Black, from the LTDB
    3. Population density, from the LTDB
    4. Rates of homicides per 100,000 residents, calculated by dividing homicide counts from multiple sources by LTDB population counts (*54*, *55*).

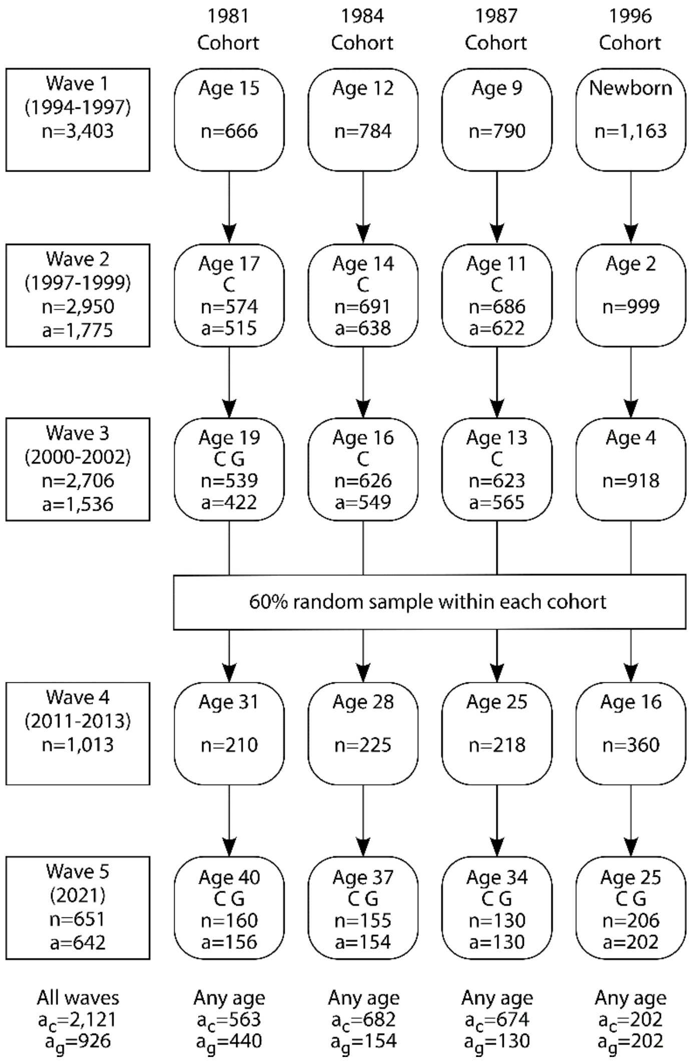

Fig. S1. PHDCN study design by age cohort and survey wave. Age is modal age of respondents at survey time for each wave. Letters indicate what questions were asked of that cohort in that wave: C is concealed carry questions and G is gun carry questions. N indicates count of respondents in each cohort surveyed at each wave. A indicates count of respondents who provided answers to gun carry (C) and gun use (G) questions. A is omitted when no questions were asked. The bottom row displays the count of respondents who provided at least one answer to either gun carry (a_C) or gun use (a_G) questions in any survey wave. Note that the sample sizes in main text analyses and in Fig. S2 correspond to the number of respondents providing answers in any wave (2,121 for carry and 926 for gun use) or in wave 5 (642 for past-year carry).

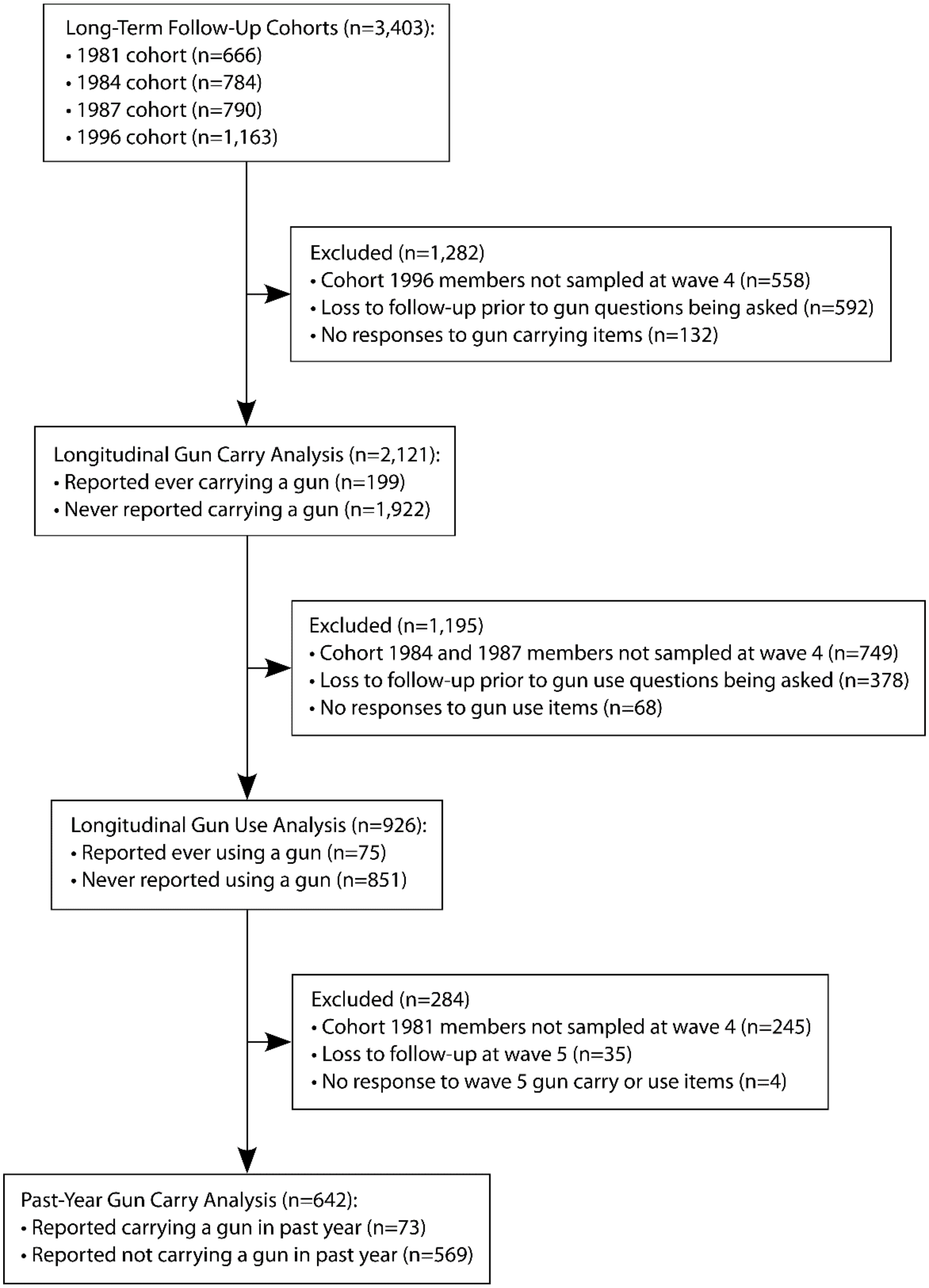

Fig. S2. Analytical samples sizes, exclusion criteria, and outcomes. Boxes connected by vertical arrows represent the baseline wave 1 sample (top box) and samples used in each analysis and contain counts of respondents reporting each outcome. Boxes connected by horizontal arrows represent excluded respondents and contain counts of respondents excluded for each reason.

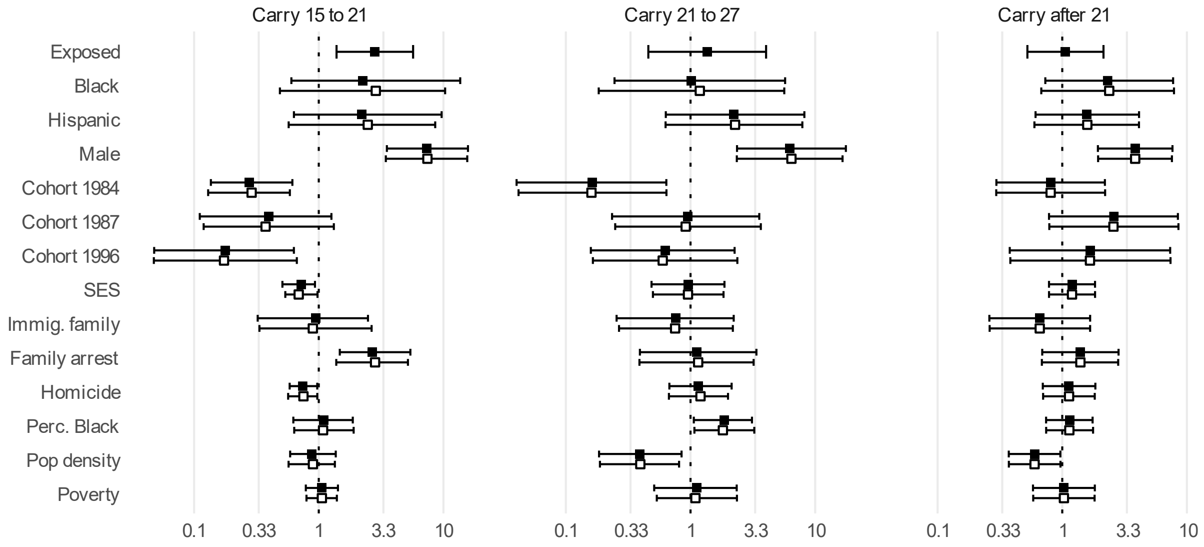

Fig. S3. Model estimates of likelihood of onset of concealed carry by age range.

Estimates are odds ratios from logistic regression with Firth correction for carry 15 to 21 and 21 to 27 and hazard ratios from Cox proportional hazards model for carry after 21. Estimates with (filled square) and without (empty square) exposure are displayed for comparison. Estimates were made using survey and attrition weights and adjusted for race, sex, age cohort, socioeconomic status, immigrant generation, history of arrest among family members, and four census tract variables: homicide rate, percent Black, population density, and percent living under the poverty line. Socioeconomic status and tract variables measured at age 12 for carry 15 to 21 and at age 18 for carry 21 to 27 and carry after 21. Main text estimates for carry 21 to 34 are derived from the carry after 21 estimates.

Table S1. Mean values of covariates across samples

	Baseline (Wave 1)	Gun Carry	Gun Use	Past-Year (Wave 5)
Black	0.35	0.36	0.35	0.34
Hispanic	0.49	0.48	0.46	0.44
Male	0.51	0.50	0.45	0.45
Cohort 1981	0.20	0.27	0.48	0.24
Cohort 1984	0.23	0.32	0.17	0.24
Cohort 1987	0.23	0.32	0.14	0.20
Cohort 1996	0.34	0.10	0.22	0.31
SES		0.00	0.21	0.33
Immigrant family	0.41	0.41	0.40	0.38
Family arrest	0.30	0.30	0.30	0.27
Homicide		30.43	29.14	24.93
Percent Black		0.34	0.33	0.31
Population density		7,798	7,234	6,839
Poverty		0.20	0.20	0.18
N	3,403	2,121	926	642

Table S1 shows unweighted mean values for covariates at baseline (wave 1) and in each analysis sample (i.e., gun carry, gun use, and past-year). Childhood *SES* is measured at wave 3 for the 1996 cohort and wave 1 for other cohorts and census tract *Homicide*, *Percent Black*, *Population density*, and *Poverty* are measured at age 12. *SES* and age 12 neighborhood measures are excluded at baseline because they were not measured until later waves for most respondents. Note that cohort composition varies across waves due to survey design (see Fig. S1).

Table S2. Self-reported arrested by age of concealed carry onset

	Age when first carried a gun		
Age of first arrest	Adolescence	Adulthood	Never carried
Adolescence	74%	31%	21%
Adulthood	4%	30%	10%
Ever Arrested	78%	61%	31%
